# Workforce in the pharmaceutical services of the primary health care of SUS, Brazil

**DOI:** 10.11606/S1518-8787.2017051007110

**Published:** 2017-09-22

**Authors:** Marselle Nobre Carvalho, Juliana Álvares, Karen Sarmento Costa, Augusto Afonso Guerra, Francisco de Assis Acurcio, Ediná Alves Costa, Ione Aquemi Guibu, Orlando Mario Soeiro, Margô Gomes de Oliveira Karnikowski, Silvana Nair Leite

**Affiliations:** I Departamento de Saúde Coletiva. Universidade Estadual de Londrina. Londrina, PR, Brasil; II Departamento de Farmácia Social. Faculdade de Farmácia. Universidade Federal de Minas Gerais. Belo Horizonte, MG, Brasil; III Núcleo de Estudos de Políticas Públicas. Universidade Estadual de Campinas. Campinas, SP, Brasil; IV Programa de Pós-Graduação em Saúde Coletiva. Departamento de Saúde Coletiva. Faculdade de Ciências Médicas. Universidade Estadual de Campinas. Campinas, SP, Brasil; V Programa de Pós-Graduação em Epidemiologia. Faculdade de Medicina. Universidade Federal do Rio Grande do Sul. Porto Alegre, RS, Brasil; VI Instituto de Saúde Coletiva. Universidade Federal da Bahia. Salvador, BA, Brasil; VII Faculdade de Ciências Médicas. Santa Casa de São Paulo. São Paulo, SP, Brasil; VIII Faculdade de Ciências Farmacêuticas. Pontifícia Universidade Católica de Campinas. Campinas, SP, Brasil; IX Faculdade de Ceilândia. Universidade de Brasília. Brasília, DF, Brasil; X Departamento de Ciências Farmacêuticas. Universidade Federal de Santa Catarina. Florianópolis, SC, Brasil

**Keywords:** Pharmaceutical Services, manpower, Patient Care Team, Workers, Primary Health Care, Health Services Research, Unified Health System, Assistência Farmacêutica, recursos humanos, Equipe de Assistência ao Paciente, Trabalhadores, Atenção Primária à Saúde, Pesquisa sobre Serviços de Saúde, Sistema Único de Saúde

## Abstract

**OBJECTIVE:**

To characterize the workforce in the pharmaceutical services in the primary care of the Brazilian Unified Health System (SUS). METHODS This is a cross-sectional and quantitative study, with data from the *Pesquisa Nacional sobre Acesso, Utilização e Promoção do Uso Racional de Medicamentos – Serviços, 2015* (PNAUM – National Survey on Access, Use and Promotion of Rational Use of Medicines – Services, 2015). For the analysis, we considered the data stratification into geographical regions. We analyzed the data on workers in the municipal pharmaceutical services management and in the medicine dispensing units, according to the country’s regions. For the statistical association analysis, we carried out a Pearson correlation test for the categorical variables.

**RESULTS:**

We analyzed 1,175 pharmacies/dispensing units, 507 phone interviews (495 pharmaceutical services coordinators), and 1,139 professionals responsible for medicine delivery. The workforce in pharmaceutical services was mostly constituted by women, aged from 18 to 39 years, with higher education (90.7% in coordination and 45.5% in dispensing units), having permanent employment bonds (public tender), being for more than one year in the position or duty, and with weekly work hours above 30h, working both in municipal management and in medicine dispensing units. We observed regional differences in the workforce composition in dispensing units, with higher percentage of pharmacists in the Southeast and Midwest regions.

**CONCLUSIONS:**

The professionalization of municipal management posts in primary health care is an achievement in the organization of the workforce in pharmaceutical services. However, significant deficiencies exist in the workforce composition in medicine dispensing units, which may compromise the medicine use quality and its results in population health.

## RESUMO

**OBJETIVO:**

Caracterizar a força de trabalho da assistência farmacêutica na atenção básica do Sistema Único de Saúde.

**MÉTODOS:**

Trata-se de estudo transversal de abordagem quantitativa com dados da Pesquisa Nacional sobre Acesso, Utilização e Promoção do Uso Racional de Medicamentos – Serviços, 2015. Para a análise, foi considerada a estratificação dos dados por região geográfica. Foram analisados dados sobre os trabalhadores na gestão da assistência farmacêutica municipal e nas unidades de dispensação de medicamentos segundo regiões do país. Para a análise de associação estatística foi realizado teste de correlação Pearson para as variáveis categóricas.

**RESULTADOS:**

Foram observadas 1.175 farmácias/unidades dispensadoras, 507 entrevistas telefônicas (495 coordenadores da assistência farmacêutica), e 1.139 responsáveis pela entrega de medicamentos. A força de trabalho na assistência farmacêutica era predominantemente composta por mulheres, na faixa etária de 18 a 39 anos, com formação superior (90,7% na coordenação e 45,5% na unidade de dispensação), com vínculo empregatício efetivo (concursado), há mais de um ano no cargo ou atividade e jornada de trabalho semanal superior a 30 horas, tanto na gestão municipal quanto nas unidades de dispensação de medicamentos. Foram observadas diferenças regionais na composição da força de trabalho nas unidades de dispensação; nas regiões Sudeste e Centro-Oeste havia maior proporção de farmacêuticos nestes serviços.

**CONCLUSÕES:**

A profissionalização das funções de gestão municipal na atenção básica é uma conquista na organização da força de trabalho da assistência farmacêutica. No entanto, há importantes deficiências na composição da força de trabalho nas unidades de dispensação de medicamentos que devem comprometer a qualidade do uso dos medicamentos e seus resultados na saúde da população.

## INTRODUCTION

Since its implementation in the late 1980s, the Brazilian Unified Health System (SUS) expanded the number of work posts in all health care levels, especially in primary health care (PHC). The expansion and diversification of health workforce occurred with an increase in the number of doctors and nurses and the inclusion of new professional categories, such as physical therapists, pharmacists, nutritionists, speech and language therapists, physical education professionals, among others^6^.

SUS holds more than 60% of the health sector facilities, assists about 80% of the population, and absorbs about 80% of the sector’s workforce, which represents almost two millions jobs in the country. It is estimated that 52% of nurses, 44% of doctors, 27% of dentists, 11% of pharmacists, and 10% of psychologists are civil servants^13^.

With the objective of reorganizing SUS and meeting the principles of integrality, equity, and universality of health care, and having PHC as an organizer of the health services network, the Brazilian Ministry of Health implemented the *Programa Saúde da Família* (PSF – Brazilian Family Health Program) in 1994, oriented to the reduction of maternal and infant mortality, especially in the North and Northeast of the country. In 2006, the government published the Política Nacional de Atenção Básica (PNAB – National Primary Health Care Policy), which transformed the PSF into the *Estratégia Saúde da Família* (ESF – Brazilian Family Health Strategy), reaffirming its major role in the reorganization of PHC^14^.

Differing form the Family Health teams, comprised by doctors, nurses, dental surgeons, oral health assistants or technicians, nursing assistants or technicians, and community health agents, the *Núcleos de Apoio à Saúde da Família* (NASF – Family Health Support Centers) teams, implemented more recently, can be constituted by numerous professional categories, among which the pharmacists are^6^ ^,^ ^15^.

Around the world, as well as in Brazil, the publishing of the World Health Report 2006 highlighted the discussion on the health work in all sectors, including pharmacy, by pointing out the shortage of workers in the health sector and the unequal workforce distribution^9^.

Most Brazilian studies on health workforce refer to the nursing category in its diverse work scenarios^10^. Compared to other higher education professional categories, there is still few studies, among which is the one by Carvalho et al.^6^ on the expansion and diversification of the workforce in SUS’s Primary Health Care. In this study, the authors point out NASF as an important strategy for the insertion of other professional categories into PHC, especially physical therapists, psychologists, social assistants, nutritionists, and pharmacists.

During the implementation and organization of SUS, changes in funding and access to essential materials also occurred, notably the National Drug Policy, in 1998. The *Política Nacional de Assistência Farmacêutica* (PNAF – National Policy of Pharmaceutical Services), published by the Resolution no. 338 of the National Health Council, in 2004, reaffirmed the pharmaceutical services as an integral part of the health system, the specific funding block creation oriented towards guaranteeing the policy’s execution in the country, and the need for human resources development for pharmaceutical services recognition^4^.

In 1998, the report from the consultative council of the WHO is a milestone for the reorientation of the pharmacist actions on health systems, shifting the actions focus from the medicines to the user. Thus, the pharmacist was recognized as the best-qualified professional to conduct actions for improving the access and promoting the rational use of medicines, being essential to organize the necessary services to the complete development of pharmaceutical services^1^.

In Brazil, studies show that, despite of the pharmaceutical category growth as a workforce in PHC, in a 75% ratio from 2008 to 2013^6^, technician professionals and other professional categories act on the public and private medicine dispensing services^5^ ^,^ ^18^.

In this context, to know the pharmaceutical services’ workforce in primary health care in indispensable to the assessment of achievements and challenges in availability management, access, and medicines’ use in the country. This would allow the preparing and following of public policies, meeting the professionals’ real educational and qualification needs for the development of pharmaceutical services and for the achievement of better health outcomes with pharmaceutical services.

The *Pesquisa Nacional sobre Acesso, Utilização e Promoção do Uso Racional de Medicamentos – Serviços, 2015* (PNAUM – National Survey on Access, Use and Promotion of Rational Use of Medicines – Services, 2015) aimed to characterize the organization of pharmaceutical services in SUS primary health care – for promoting the access and rational use of medicines –, as well as to identify and discuss the issues that interfere in the municipal pharmaceutical services consolidation.

This study is part of PNAUM – Services and aimed to characterize the pharmaceutical services workforce in the Brazilian primary health care networks.

## METHODS

PNAUM is a cross-sectional, exploratory, and evaluative study, composed of an information gathering in a representative sample of primary health care services, in cities of Brazilian regions. Several study populations were considered in the sampling plan, with samples stratified by regions, which constitute the study domains^2^. The Brazilian Ministry of Health’s Decree no. 2,077, of September 17, 2012, instituted the survey.

The sampling considered the cities’ regional representation: capitals; bigger cities, selecting the 0.5% biggest cities in the region; and smaller cities, totaling 120 cities by region. In each city, primary health care services were sampled according to the total number of services in that city. For this study, we analyzed the face-to-face interviews made with the professionals responsible for the medicines’ delivery in the SUS primary health care services, in addition to the observations of the pharmaceutical services facilities of all sampled services and phone interviews with those responsible for municipal pharmaceutical services (one in each city). The interview scripts comprised the pharmaceutical services organization in each city and health service, including professional practice, available structures, and work processes. The observation script for the health services facilities comprised a checklist and photographical register that were applied by trained professionals. The research team collectively built, pretested, and validated all survey instruments in health services in the state of Minas Gerais. The interviewers received specific training for each applied instrument. The complete survey methodology description is available at Álvares et al.^2^

The data were collected from July to December 2014. For the analysis, we considered the data stratification by geographical regions.

All data were analyzed with the assistance of the SPSS software, version 22, extracting the frequencies of the study variables. All analyses considered sample weights and the analysis plan structure for complex samples. For the statistical association analysis, the Pearson correlation test was carried out for categorical variables. The significance level adopted was p < 0.05. The results showed representativeness for the Brazilian geographical regions.

The participants signed the informed consent form. PNAUM – Services was approved by the National Research Ethics Committee of the National Health Council, by Opinion no. 398,131/2013.

## RESULTS

We analyzed 1,175 pharmacies/dispensing units, 507 phone interviews (495 pharmaceutical services coordinators), and 1,139 professionals responsible for medicines delivery.

The workforce in the municipal pharmaceutical services management was mostly composed of women. The Northeast was the only region where men occupy 50.1% (95%CI 39.2-61.0) of the coordination posts (Table 1).

**Table 1:** Characterization of municipal pharmaceutical services managers, Brazil and regions. National Survey on Access, Use and Promotion of Rational Use of Medicines – Services, 2015. (n = 507)

Variable	North	Northeast	Midwest	Southeast	South	Brazil
					
	% (95%CI)	% (95%CI)	% (95%CI)	% (95%CI)	% (95%CI)	% (95%CI)
Sex						
Female	64.4 (54.0–73.6)	49.9 (39.9–60.8)	67.3 (57.3–75.9)	63.2 (53.1–72.3)	73.2 (63.7–81.0)	62.0 (56.9–66.9)
Male	36.6 (26.4–46.0)	50.1 (39.2–61.0)	32.7 (24.1–42.7)	36.8 (27.7–46.9)	26.8 (19.0–36.3)	38.0 (33.1–43.1)
Age group (years)						
18 to 39	75.1 (64.8–83.2)	66.3 (55.1–75.9)	84.0 (75.2–90.1)	69.6 (59.5–78.1)	72.5 (62.9–80.4)	70.9 (65.8–75.5)
40 to 59	24.9 (16.8–35.2)	29.9 (20.7–40.9)	16.0 (9.9–24.8)	30.3 (21.9–40.4)	27.5 (19.6–37.1)	27.9 (23.4–33.0)
≥ 60	–	3.8 (1.2–11.4)	–	1.0 (0–5.0)	–	1.2 (0.4–3.5)
Employment bond						
Permanent	37.5 (28.2–47.9)	39.9 (29.7–51.1)	45.5 (35.8–55.5)	63.2 (53.1–72.3)	76.8 (67.6–84.0)	56.0 (51.0–60.9)
Commission-based	35.6 (26.4–46.0)	34.6 (25.0–45.7)	25.5 (17.7–35.1)	14.8 (9.0–23.4)	10.0 (5.5–17.6)	22.0 (18.1–26.5)
Other	26.9 (18.7–37.0)	25.5 (17.0–36.2)	29.1 (20.9–39.0)	22.0 (14.8–31.5)	13.2 (7.9–21.3)	22.0 (18.0–26.6)
Workweek (weekly hours)						
≤ 30	45.3 (35.3–55.7)	62.4 (51.3–72.4)	22.9 (15.5–32.4)	27.9 (20.0–37.4)	27.9 (20.0–37.4)	39.1 (34.4–44.0)
> 30	54.7 (44.3–64.7)	37.6 (27.6–48.7)	77.1 (67.6–84.5)	71.8 (61.9–79.9)	72.1 (62.6–80.0)	60.9 (56.0–65.6)
Time in position (months)						
≤ 12	38.4 (28.8–49.0)	28.6 (19.6–39.4 )	39.4 (30.1–49.5)	24.4 (16.8–34.1)	23.3 (16.0–32.5)	37.7 (23.3–32.5)
> 12	61.6 (51.0–71.2)	71.4 (60.4–80.4)	60.6 (50.5–69.9)	75.6 (65.9–83.2)	76.7 (67.5–84.0)	72.3 (67.5–76.7)

Source: PNAUM – Services, 2015. Permanent: public tender or transferred servant. p-values (sex) = 0.008; p (age group) = 0.022; p (employment bond) < 0.001; p (weekly workhours) < 0.001; p (time in position) = 0.167.

Concerning education level, most cities had managers with higher education degree. The Northeast region stands out by two important aspects: 22.2% (95%CI 14.4–32.8) of the cities had managers with specialization (*lato sensu*) and 3.8% (95%CI 1.2–11.4) had managers with master’s or doctor’s degree (*stricto sensu*) in any knowledge area. All regions showed predominance of pharmacists (> 80.0%) as coordinators of municipal pharmaceutical services (Table 1).

Concerning the type of employment bond, in the Southeast and South regions, cities with permanent managers predominated, whereas in other regions there was a certain similarity between permanent and commission-based workers. All regions showed prevalence of time in position higher than 12 months (> 70.0%). Excluding the Northeast, the managers of the other regions had weekly work hours higher than 30h (Table 1).

**Figure 1:**
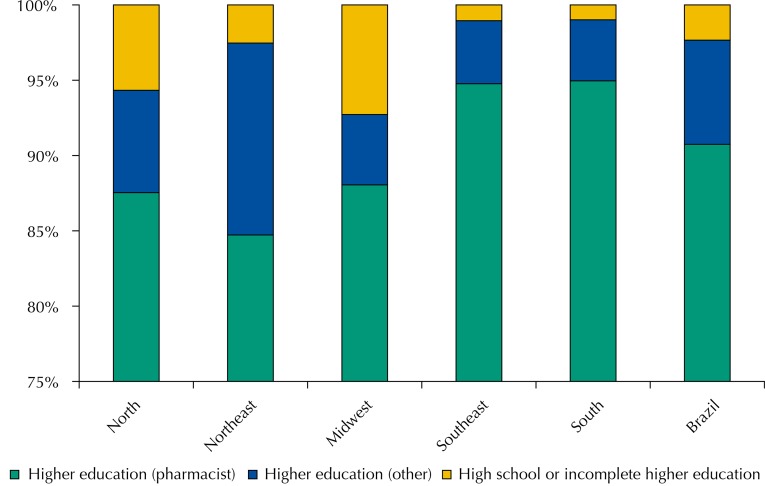
Education level and profession of the pharmaceutical services managers in the primary health care, Brazil and regions. National Survey on Access, Use and Promotion of Rational Use of Medicines – Services, 2014. (n = 507).

Source: PNAUM – Services, 2015. p-value = 0.023.

Pharmacists predominated in the composition of the management of municipal pharmaceutical services in all regions. More than 90.0% of the Brazilian cities had pharmacists in the coordination of pharmaceutical services (Figure 1).

Women were the majority of workers in the medicine dispensing units of primary health care health services in all regions, notably in the Northeast and South, where women predominated in more than 80.0% of the cities (Table 2).

**Table 2:** Characterization of workers in the medicine dispensing units in SUS primary health care, Brazil and regions. National Survey on Access, Use and Promotion of Rational Use of Medicines – Services, 2015. (n = 1,139)

Variable	North	Northeast	Midwest	Southeast	South	Brazil
					
	% (95%CI)	% (95%CI)	% (95%CI)	% (95%CI)	% (95%CI)	% (95%CI)
Sex						
Female	77.5 (70.6–83.2)	80.9 (72.2–87.3)	68.8 (51.6–82.0)	69.7 (52.8–82.6)	87.4 (81.1–91.9)	77.4 (71.2–82.6)
Male	22.5 (16.8–29.4)	19.1 (12.7–27.8)	31.2 (18.0–48.4)	30.3 (17.4–47.2)	12.6 (8.1–18.9)	22.6 (17.4–28.8)
Age group (years)						
18 to 39	59.3 (52.9–65.4)	68.6 (57.0–78.3)	71.1 (59.6–80.4)	51.0 (37.9–63.9)	66.0 (50.4–78.7)	61.9 (55.0–68.3)
40 to 59	38.4 (32.3–44.9)	29.1 (20.4–39.6)	25.5 (17.1–36.2)	45.9 (33.9–58.4)	32.5 (19.9–48.2)	35.6 (29.6–42.1)
≥ 60	2.3 (1.2–4.3)	2.3 (0.8–6.2)	3.4 (0.7–15.8)	3.1 (1.6–5.9)	1.5 (0.3–7.2)	2.5 (1.5–4.0)
Employment bond						
Permanent	43.1 (33.9–52.7)	42.7 (30.7–55.7)	61.8 (45.0–76.2)	66.2 (49.4–79.7)	76.2 (56.2–88.8)	57.0 (49.3–64.4)
Contract	53.4 (43.3–63.2)	48.5 (36.4–60.8)	33.0 (19.7–49.8)	15.9 (9.8–24.7)	12.2 (6.3–22.4)	31.5 (25.7–37.9)
Outsource	0.5 (0.1–3.5)	3.0 (1.2–7.4)	2.8 (0.6–11.8)	7.6 (3.5–15.6)	1.4 (0.5–4.0)	4.0 (2.3–6.7)
Commission-based	0.9 (0.2–3.7)	4.0 (1.4–10.5)	1.5 (0.4–5.5)	9.5 (2.0–34.6)	9.5 (2.2–31.9)	6.2 (2.6–14.0)
Other	2.1 (1.1–4.1)	1.8 (0.6–4.9)	0.9 (0.2–3.9)	0.8 (0.2–3.2)	0.9 (0.2–3.1)	1.3 (0.7–2.4)
Workweek (weekly hours)						
≤ 30	43.8 (31.2–57.2)	32.5 (20.2–47.8)	29.5 (16.2–47.6)	39.1 (24.7–55.5)	34.8 (18.9–55.0)	35.8 (28.1–44.3)
> 30	56.2 (42.8–68.8)	67.5 (52.2–79.8)	70.5 (52.4–83.8)	60.9 (44.5–75.3)	65.2 (45.0–81.1)	64.2 (55.7–71.9)
Experience period (months)						
≤ 12	10.6 (6.0–18.0)	11.0 (6.5–18.1)	8.1 (2.6–22.5)	8.8 (3.8–18.8)	12.7 (6.2–24.0)	10.4 (7.4–14.4)
> 12	89.4 (82.0–94.0)	89.0 (81.9–93.5)	91.9 (77.5–97.4)	91.2 (81.2–96.2)	87.3 (76.0–93.8)	89.6 (85.6–92.6)

Source: PNAUM – Services, 2015. Assistant: pharmacy or nursing assistant. Permanent: public tender or transferred servant. p-values (sex) = 0.087; p (age group) = 0.139; p (employment bond) = 0.001; p (weekly work hours) = 0.803; p (experience period) = 0.886.

Concerning the workers’ age group, North and Southeast showed similar patterns: the groups from 18 to 39 years and from 40 to 59 years were virtually equivalent, i.e., the workforce was almost equally distributed in these age groups (Table 2).

In the Midwest, Southeast, and South, more than 60.0% of the cities had permanent workers, whereas in the North and Northeast, the percentage of public permanent and contracted workers was proportional. The Southeast, presented a significant percentage of cities with outsourced workers in the medicine dispensing units (7.6%; 95%CI 3.5–15.6). Regarding the experience period and weekly work hours, all regions showed prevalence of more than one year in medicine dispensing and 30h weekly work hours (Table 2).

While the coordination was mostly constituted by women with higher education, especially graduated in Pharmacy, the medicine delivery to users was done by women with high school education level, essentially technicians or nursing assistants (Tables 1 and 2).

**Figure 2:**
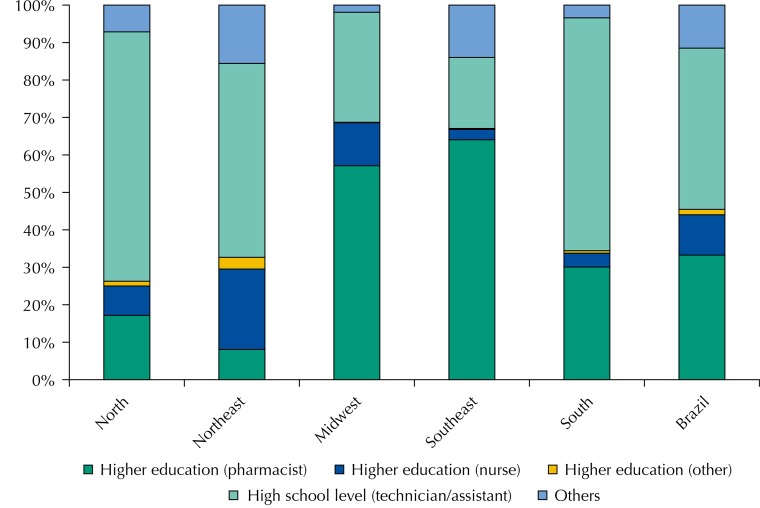
Education level and profession of workers in the dispensing units of the primary health care, Brazil and regions. National Survey on Access, Use and Promotion of Rational Use of Medicines – Services, 2015. (n = 1,139).

Source: PNAUM – Services, 2015. p-values < 0.001.

Figure 2 refers to the workforce in the medicine dispensing units of the health services in SUS primary health care: it was mainly composed of technicians or nursing assistants (n = 631; 43.0%; 95%CI 36.0–50.4; p < 0.001), followed by pharmacists (n = 291; 33.3%; 95%CI 26.4–40.9; p < 0.001).

The worker’s education level and qualification in the medicine delivery/dispensing units varied significantly according to the region. In the North, South, and Northeast regions, workers with high school education level (mostly technicians or nursing assistants) predominated, while the Midwest and Southeast had a higher number of pharmacists (57.1%, 95%CI 41.0–71.9; 64.1%, 95%CI 45.9–78.9; p < 0.001 respectively).

Besides pharmacists, nurses also performed activities in this type of unit (n = 108; 10.8%; 95%CI 7.2–15.7). In the Northeast, 21.5% (95%CI 12.6–34.0) of the cities had a nurse as a professional who delivered medicines in the primary care health units (Figure 2).

## DISCUSSION

All cities that participated in PNAUM – Services had at least one pharmacist in the municipal health network. They were concentrated in the municipal management activities and had been in the position for more than one year, showing a movement towards the consolidation of this sector in the municipal management structure.

PNAUM – Services data show that the pharmaceutical services workforce in the primary health care was mostly composed of women, aged from 18 to 39 years, with permanent employment bond, having been in the position for more than one year or having weekly work hours above 30h. The education level, in turn, varied according to the activity: higher education degree in coordination posts and technician or assistant level in the health units.

The pronounced presence of women in the coordination activities of the municipal pharmaceutical services can be a result of the widening of access to higher education in Pharmacy and of the increase in the number of women in the pharmaceutical market. Women are also the majority among the dispensing units’ workers, whose predominant education level was high school (technician or assistant).

There are many explanations to the hegemonic presence of women in the workforce, among which notably is the increase of women in society and university. For some authors, the intensification of women participation in the market is also because they are a cheaper workforce and accept to work in precarious conditions. In this context, the literature highlights the polarization of Brazilian female workers, in which, in one side, there are women with higher education and relatively high salaries considering the female workers group, and, on the other side, there are women with low qualification, low salaries, and lesser social recognition^7^.

In the health area, besides the aforementioned reasons, the traditional “female jobs,” or the ones historically and socially identified as “female-like” explain the major presence of woman as nurses, nutritionists, psychologists, social assistants, and midwives. However, we can already observe the insertion of women in male-dominated areas, such as medicine^19^.

Indeed, the literature points out the expansion of female participation in male university careers, e.g., medicine, dentistry, and veterinary medicine; and the rising insertion of women in the health area market, where they already represents more than 70.0% of the workforce. In many undergraduate courses in health, including Pharmacy courses, women already occupy more than half of the openings. Among the high school level professionals, the feminization tendency seems stronger, representing 86.9% of technicians and nursing assistants^7^ ^,^ ^12^.

Wermelinger et al.^19^ bring up an interesting reflection on the most feminine job in the health area: nursing. In a context in which technicality is well established and even explains the male work valuing, the nursing work is disqualified by being a mostly female job. Its activities increasingly demand the mastering of new technologies, including hard ones, both within care and management. However, the nursing work social representation is associated to more human and less technical aspects of health care, which is mistakenly translated as less valuable work. This reflection is important to the results presented in this study, which clearly show the presence of women with higher education in management positions and of women with technician education in medicine dispensing.

Overall, most of the management and health care workers have permanent employment bond (56.0% and 57.0%, respectively), i.e., they entered public administration by public tenders. Differing from the national outcome, the North and Northeast regions showed proportional distribution between permanent and commission-based workers in the municipal coordination of pharmaceutical services and predominance of contracted workers in dispensing units (53.4% and 48.5%, respectively).

In this context, it is important to highlight that around 35.0% of dispensing units’ workers are contracted or outsourced, which can indicate a tendency of flexibilization or even precarization of labor relations.

While permanent workers are the majority in the Midwest, Southeast, and South regions, the North and Northeast regions stand out by the predominance of contracted workers. Outsourcing represents 7.6% of the employment bonds in the Southeast region, probably due to many factors, among which are the limitations imposed by the Fiscal Responsibility Law.

Outsourcing – one of the main flexibilization expressions – is an especial way of privatization that public administration uses to partially transfer the responsibility for the production of some services, by hiring third parties. Although it is an efficient way of ensuring the compliance with the Fiscal Responsibility Law, the advantages and/or disadvantages of flexibilization depend on the point of view, for it is important to point out two tendencies in this process: the job protection deregulation and the precariousness of labor relations, with consequent distinction of job and salary and a tendency to the fragmentation of labor relations ^3^ ^,^ ^11^.

Although other professional categories can take part in the management, structuring, and organization, pharmacists (90.7%) perform the municipal coordination of pharmaceutical services in all country’s regions, regionally varying from 84.7% in the Northeast to 95.0% in the South. This is probably due to many factors: from the existence of sector guiding policies, such as PNAF, to the pharmaceutical services reorientation actions in SUS and the structuring of pharmaceutical services in the cities^4^.

According to the world workforce report on Pharmacy^9^, in countries with low *per capita* income (e.g. Republic of the Congo and Haiti), the lack of pharmacists resulted in the dependence on high school level workers. In high *per capita* income countries (e.g. Australia, United Kingdom, and Japan), pharmacists are the majority in the workforce composition. The participation of high school level workers, usually regulated and qualified professionals to assist the pharmacist, varies from 43.2% in Europe to 28.4% in Americas, while in the Southeast Asia it represents 67.5% of the total pharmaceutical area workforce. It is important to highlight that these outcomes refer to the work market in general, without any specific approach to the primary health care services in the analyzed countries.

In Brazil, the scenario seems favorable concerning the number of pharmacists. According to the Brazilian Ministry of Labor and Social Security^a^, there was 5.4 pharmacists per 10,000 inhabitants in 2013, a number higher than in countries such as Mexico, India, and all African countries, but lower than in many European countries and in the United States of America. Considering all those who work under professional registration, in 2014, the *Conselho Federal de Farmácia* (CFF – Federal Pharmacy Council) estimated nine pharmacists for each 10,000 inhabitants^17^.

The discussion on the pharmaceutical services workforce in SUS, however, is unique and deserves observation. Between 2008 and 2013, the number of pharmacists registered in the *Unidades Básicas de Saúde* (UBS – Basic Health Units) grew 75.0% in the country. Excluding the Northeast (45.0%), the growth rates were higher than 50.0% in the other regions; two factors possibly enabled it: the implementation of the *Núcleo de Apoio à Saúde da Família* (NASF – Family Health Support Center) and the growth of pharmaceutical services in the country. Besides that, Brazil is one of the few countries who have a public pharmaceutical service model in which pharmacists coordinate all activities related to the medicine chain in government spheres, from selection to use^4^ ^,^ ^6^.

The pharmaceutical hegemony identified in the municipal management of pharmaceutical services is not reproduced in dispensing units. The high school level workers, especially technicians and nursing assistants, occupied most of the work positions (43.0%), while pharmacists represented 33,3% of the workforce present in these units. When we verified the technical responsibility for dispensing units, we verified that 43.0% of them had pharmacists in charge (varying from 18.6% in the Northeast to 72.0% in the Southeast).

Despite the high growth rates, from 50.0% to 150.0% – as in the case of speech and language therapists (55.0%), pharmacists (75.0%), physical therapists (78%), nutritionists (83%), and physical education professionals (145.0%) –, these categories still have low percentage participation in the UBS’s workforce, varying from 0.1% to 3.0%. In 2008, pharmacists represented 2.9% of all UBS registered workers^6^.

Similar to the pattern in low and high-income countries, we also observed in Brazilian regions the hegemony shifting between pharmacists and high school level workers in dispensing units. The pharmacists’ participation varied from 8.1% in the Northeast to 64.1% in the Southeast, while technicians and assistants predominated in the North (66.6%) and represented 19.0% of the workforce in the Southeast. This is probably due to the same factors identified in the world regions, among which are notably the courses offer (high and technical education), Human Development Index (HDI), among other factors^8^ ^,^ ^9^.

In fact, the Southeast region concentrates the pharmaceutical education in the country: in 2013, there were 216 Pharmacy courses (50.0%), 14,475 students, and 7,626 graduated, while the North region had 27 courses, 1,802 students, and only 862 graduated. In addition, Southeast and South have the best HDIs. In this sense, it seems that the predominance of higher education professionals (especially pharmacists) depends on the offer of higher education courses and on the conditions of health professionals’ fixation, which usually depend on the regional wealth level and development^b^ ^,^ ^c^.

It is also important to consider the type of organization of medicine dispensing units in the different regions. In the Southeast region, there is a higher concentration of centralized pharmacies and the consequent decrease in the number of dispensing units in the UBS. Therefore, the decrease in units with medicine dispensing increases the proportion of units that have pharmacists, even though the absolute number of pharmacists does not show such difference.

Generally, pharmacists have been expanding their functions and responsibilities in primary health care all around the world, clearly focusing on direct patient care. Studies on the pharmaceutical workforce carried out in the United States of America identified that service provision and patient assistance by pharmacists have increased. The sector of medicine supplier pharmacists who also care for patients went from 17.0% (2009) to 48.0% (2014). Nevertheless, the medicine suppliers’ maintenance in stable levels suggests the need to keep the pharmacists also involved in the medicine offer as an essential activity to the good performance of health services^16^.

In conclusion, the professionalization of municipal management functions in the organization of the pharmaceutical services workforce in the SUS primary health care is an achievement. However, there are significant deficiencies in the workforce composition of medicine dispensing units, which may affect the quality of the medicine use and its results on health. Despite the representative sampling from all the regions and population ranges, study limitations may have created bias, such as in the municipal dispensing units’ sampling, since it presents many possibilities of organization and distribution in different ways among each other. The information registered here were gathered from interviews with the professionals responsible for the municipal pharmaceuticals services and with workers who, during data collection, identified themselves as responsible for the medicine dispensing in the units, thus prone to interpretation bias.

## Footnotes

a Former *Ministério do Trabalho e Emprego* (MTE – Ministry of Labor and Employment). Current *Ministério do Trabalho e Previdência Social* (MTPS – Ministry of Labor and Social Security). *Relação Anual de Informações Sociais* (RAIS – Annual Report of Social Information). Available from: http://bi.mte.gov.br/bgcaged/login.php.

b Observa RH – Estação de Trabalho IMS/UERJ. SIGRAS – Sistema de Sistema de Indicadores das Graduações em Saúde. Rio de Janeiro: IMS/UERJ; 2013 [cited 2017 Jan 26]. Available from: http://www.obsnetims.org.br/sigras

c Instituto de Pesquisa Econômica Aplicada. O Índice de Desenvolvimento Humano Municipal Brasileiro 2013. Brasília (DF): PNUD, IPEA, FJP; 2013 [cited 2017 Jan 26]. (Série Atlas de Desenvolvimento Humano no Brasil 2013). Available from: http://www.ipea.gov.br/portal/images/stories/PDFs/130729_AtlasPNUD_2013.pdf
